# Pattern dynamics of the reaction-diffusion immune system

**DOI:** 10.1371/journal.pone.0190176

**Published:** 2018-01-31

**Authors:** Qianqian Zheng, Jianwei Shen, Zhijie Wang

**Affiliations:** 1 College of Information Science and Technology, Donghua University, Shanghai, Shanghai, China; 2 Institute of Applied Mathematics, Xuchang University, Xuchang, Henan, China; North University of China, CHINA

## Abstract

In this paper, we will investigate the effect of diffusion, which is ubiquitous in nature, on the immune system using a reaction-diffusion model in order to understand the dynamical behavior of complex patterns and control the dynamics of different patterns. Through control theory and linear stability analysis of local equilibrium, we obtain the optimal condition under which the system loses stability and a Turing pattern occurs. By combining mathematical analysis and numerical simulation, we show the possible patterns and how these patterns evolve. In addition, we establish a bridge between the complex patterns and the biological mechanism using the results from a previous study in Nature Cell Biology. The results in this paper can help us better understand the biological significance of the immune system.

## Introduction

For a reaction-diffusion system, the biological mechanism of pattern formation was proposed by Turing [1]. Recently, the study of pattern dynamics about secondary bifurcation, Turing bifurcation and amplitude equations, etc has attracted more attention [2–5] and be used in different fields [6–8]. It turned out that external periodic forcing and dynamical parameters affected the shape and type of patterns [9]. And research on the robustness problem with respect to biological systems had also been reported [10]. The cross-diffusion, one species affects other species in concentration gradient, had been considered in some fields [11–13]. And noise is an important way to explain synthetic systems and understand organismal phenotypes [14, 15]. Then Turing instability on networks was proposed to understand multicellular morphogenesis [16]. In addition, Turing patterns in large random networks was studied to show the significant difference with classical systems [17–19]. However, control theory was seldom used to study pattern dynamics.

Controllability and observation, which are the central concepts in control theory, are vital in designing a controller of a dynamical system [20]. Maidi and Corriou studied the controllability of a particular class of distributed parameter systems described by a partial differential equation (PDE) [21]. And the development of a mathematical model for minimizing quadratic functionals in infinite time was investigated [22], then the stability of nonlinear dynamical systems was analyzed [23, 24]. In addition, stochastic control problems have attracted considerable research interest [25–27]. Regarding immunity, the immune system provides the body with both non-specific and specific defense against pathogens and distinguishes between foreign and native species [28–30]. Wood disclosed the mechanism of the immediate response [31], and Cho studied the heritable immune system [32]. In addition, the Biological Immune System/ Human Immune System (BIS/HIS) has many desirable features, such as adaptability, robustness, homeostasis, memory, and immunity [33–36], which can be used to solve various computational problems.

The feedback control was studied, which means nonlinear control achieves significantly better attenuation of the effect of bifurcations [37]. And Soh et al. illustrated that reaction-diffusion processes was of importance in intracellular transport and control [38]. Then a control design about a heat equation to stabilize an unstable parabolic PDE was designed [39]. Moreover, the spatiotemporal dynamics of a predator-prey system was studied in presence of additional food (controller) existing for predators [40] and these patterns may be controlled by external perturbation [41]. The pattern transitions was proved to be controlled in spatial epidemics, which provided valuable insights into disease prevention and control [42]. In addition, the controllable nonlinear diffusion processes was considered [43]and the completely observable stochastic control problems for diffusion processes was dealt [44], the control of one-dimensional diffusion processes was discussed [45]. However, the controller was seldom designed to investigate the pattern formation.

Recently mathematical models is often utilized to find the main properties of biological system and explain some biological phenomenon [46]. Adhesion proteins are known to be essential for the formation of normal epithelial tissues [47]. In the absence of adhesion proteins, genes in cell will diffuse rapidly, which causes disease. In this paper, we attempt to study pattern formation under the condition of Turing instability by modern control theory to better understand the mechanism of the immune system and present a detailed process for designing a controller. Then, we considered pattern formation with/without a controller under the critical value of Turing instability which provides a new tool to study pattern dynamics by modern control theory, especially explaining the biological mechanism of pattern formation. Finally, we establish a bridge between the complex patterns and the biological mechanism and use the above results to control the proliferation of cancer cells with medicine. These results will help us understand the immune system and will provide a theoretical method to guide clinical medication for the treatment of some conditions.

The paper is organized as follows. In Section 2, we provide an immune system model and obtain the condition of Turing instability. In Section 3, we study the reaction-diffusion equation by modern control theory. In Section 4, we show the numerical analysis of the model. Finally, we summarize our results and draw conclusions.

## The model

As we all know that immune cell plays an important role in healthy body, some methods was proposed to cure some diseases by medicine. In this section, we consider a two species competition model with finite carrying capacities about cancer cell and immune cell [48] and an effective treatment was obtained by controlling the amount of a medicine. In general, it can be written as follows:

$$\begin{array}{c} \begin{array}{c} \frac{\partial P}{\partial t}=f\left(P,Q\right),\\ \frac{\partial Q}{\partial t}=g\left(P,Q\right). \end{array} \end{array}$$ (1)

where *f*(*P*,*Q*) = *αP* − *βP*^2^ − *γPQ*, *g*(*P*,*Q*) = *θQ* − *δPQ* − *ηQ*^2^, *P* is the density of cancer cells, and *Q* is the density of immune cells. The meaning of the parameters can be obtained in reference [49].

**Fig 1:**
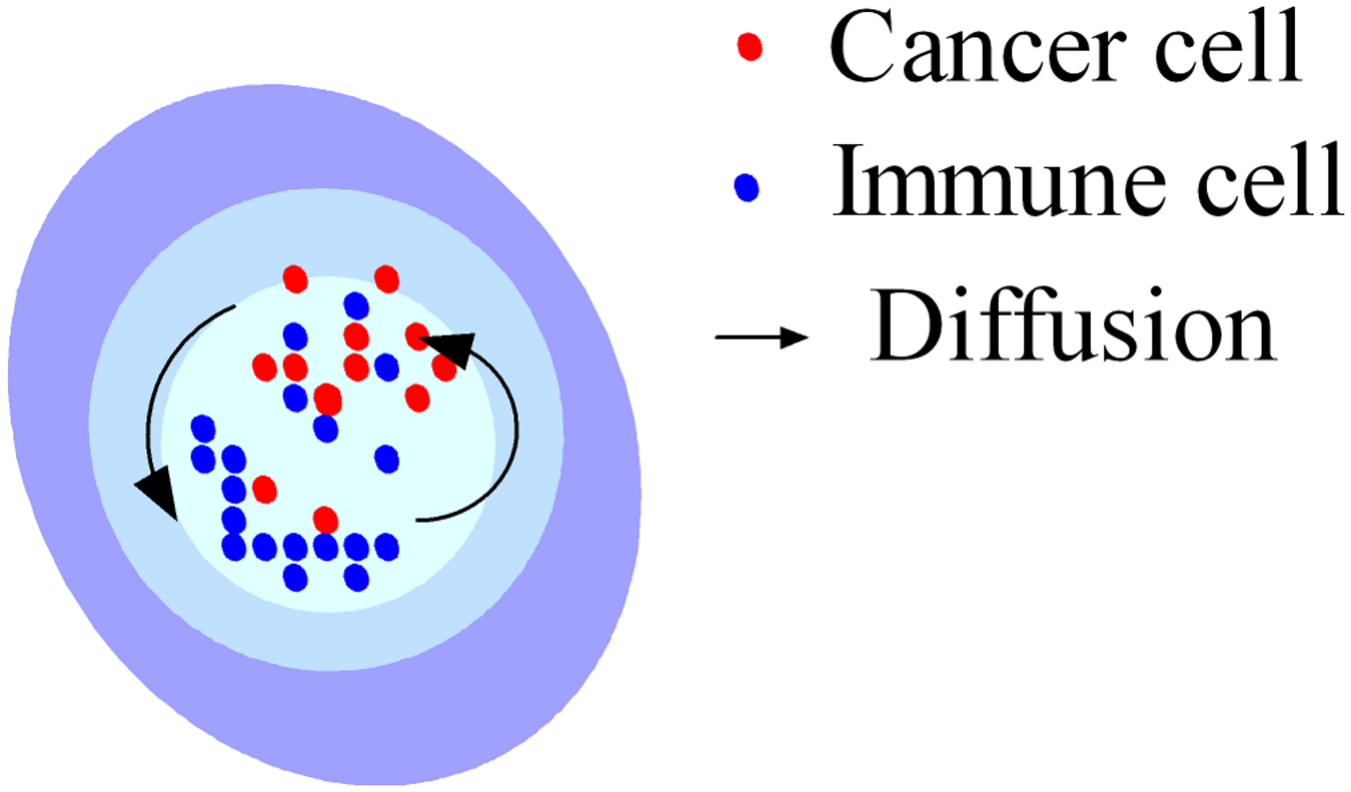
Cancer cell and immune cell with diffusion.

Diffusion is known to often be caused by the intensity of pressure and density and thus can sometimes destabilize the stable equilibrium state. Then the diffusion of cancer cell with little adhesion proteins will make organism worse and the diffusion of immune cell also occurs to protect organism in Fig 1. Therefore, we should consider the effect of diffusion on the model. In this paper, we modified the system (1), added the diffusion term to the system and obtained a reaction-diffusion system with zero boundary conditions as follows:

$$\begin{array}{c} \begin{array}{c} \frac{\partial P}{\partial t}=f\left(P,Q\right)+d_{1}\nabla^{2}P,\\ \frac{\partial Q}{\partial t}=g\left(P,Q\right)+d_{2}\nabla^{2}Q. \end{array} \end{array}$$ (2)

Here, we take the equilibrium $\left(P_{0},Q_{0}\right)=\left(\frac{\gamma \theta -\eta \alpha }{\gamma \delta -\eta \beta },\frac{\alpha \delta -\beta \theta }{\gamma \delta -\eta \beta }\right)$, which satisfies *f*(*P*_0_, *Q*_0_) = 0, *g*(*P*_0_, *Q*_0_) = 0.

And it is easy to know that the Jacobian matrix at equilibrium (*P*_0_, *Q*_0_) is as follows:

$$\begin{array}{c} A=(\begin{array}{cc} a_{11} & a_{12}\\ a_{21} & a_{22} \end{array}), \end{array}$$ (3)

where *a*_11_ = *α* − 2*βP*_0_ − *γQ*_0_, *a*_12_ = −*γP*_0_, *a*_21_ = −*δQ*_0_, *anda*_22_ = *θ* − *δP*_0_ − 2*ηQ*_0_.

Then the standard form of the linear system of (2) can be written

$$\begin{array}{c} \frac{\partial }{\partial t}(\begin{array}{c} P\\ Q \end{array})=A(\begin{array}{c} P\\ Q \end{array})+D\nabla^{2}(\begin{array}{c} P\\ Q \end{array}). \end{array}$$ (4)

In the standard way, we assume that (*P*, *Q*) takes the following form in Fourier space:

$$\begin{array}{c} (\begin{array}{c} P\\ Q \end{array})=(\begin{array}{c} c_{1}^{k}\\ c_{2}^{k} \end{array})e^{\lambda_{k}t+ikr}\left(k=1,2,3\right). \end{array}$$

Substituting the above formulation into (4) provides the characteristic equation

$$\begin{array}{c} |\begin{array}{cc} \lambda_{k}-a_{11}+k^{2}D_{1} & -a_{12}\\ -a_{21} & \lambda_{k}-a_{22}+k^{2}D_{2} \end{array}|=\lambda_{k}^{2}-Tr_{k}\lambda +\delta \left(k^{2}\right)=0, \end{array}$$

where

$$\begin{array}{c} Tr_{k}=a_{11}+a_{22}-k^{2}\left(D_{1}+D_{2}\right),\\ \delta \left(k^{2}\right)=a_{11}a_{22}-a_{12}a_{21}-\left(a_{11}D_{2}+a_{22}D_{1}\right)k^{2}+D_{1}D_{2}k^{4}. \end{array}$$

and the roots of the characteristic equation are

$$\begin{array}{c} \lambda_{k}=\frac{1}{2}\left(Tr_{k}\pm \sqrt{Tr_{k}^{2}-4\delta_{k}}\right). \end{array}$$

Finally, we get the critical value $k_{c}^{2}=\frac{a_{11}D_{2}+a_{22}D_{1}}{2D_{1}D_{2}}$ and a necessary condition of Turing instability

$$\begin{array}{c} \delta \left(k_{c}^{2}\right)=a_{11}a_{22}-a_{12}a_{21}-\left(a_{11}D_{2}+a_{22}D_{1}\right)k^{2}+D_{1}D_{2}k^{4}<0. \end{array}$$

Clearly, we can validate the result in Fig 1 by selecting the appropriate parameter.

## Control effort

It is well known that the elimination of cancer cells relies on not only immune cells but also medicament control. In this section, a controller of adjuvant therapy will be designed [50] based on the system 4. And the reaction-diffusion system with a controller can be written

$$\begin{array}{c} \frac{d}{dt}(\begin{array}{c} P\\ Q \end{array})=\left(A-Dk^{2}\right)(\begin{array}{c} P\\ Q \end{array})+Bu,R=C(\begin{array}{c} P\\ Q \end{array}). \end{array}$$ (5)

where *Dk*^2^ is obtained by substituting the type of solution in Fourier space into the Laplace operator and rewriting the type of solution.

In general, the performance indicator functional can be written as

$$\begin{array}{c} J=\frac{1}{2}\int_{0}^{\infty }\left(R^{T}MR+u^{T}Nu\right)dt, \end{array}$$

where *M* = *C*^*T*^*M*_1_*C* is positive semidefinite, *N* is positive definite and *J* minimizes the output and controller.

In order to derive the control function in the following section, we denote

$$\begin{array}{c} x=(\begin{array}{c} P\\ Q \end{array}), \end{array}$$ (6)

and

$$\begin{array}{c} \begin{array}{c} \dot{x}=Ax+Bu,\\ R=Cx. \end{array} \end{array}$$ (7)

where *A* = *A* − *Dk*^2^.

To solve the optional problem, we introduce the Hamilton function

$$\begin{array}{c} \begin{array}{c} H=\frac{1}{2}\left(x^{T}Mx+u^{T}Nu\right)+\lambda^{T}\left(Ax+Bu\right). \end{array} \end{array}$$ (8)

and have the control function

$$\begin{array}{c} \begin{array}{c} \frac{\partial H}{\partial u}=Nu+B^{T}\lambda =0. \end{array} \end{array}$$ (9)

Then,

$$\begin{array}{c} u=-N^{-1}B^{T}\lambda . \end{array}$$

and co-state function

$$\begin{array}{c} \begin{array}{c} \dot{\lambda }=-\frac{\partial H}{\partial x}=-Mx-A^{T}\lambda . \end{array} \end{array}$$ (10)

Assume

$$\begin{array}{c} \lambda =Lx. \end{array}$$

and

$$\begin{array}{c} u=-N^{-1}B^{T}\lambda =-N^{-1}B^{T}Lx=-Kx. \end{array}$$

where

$$\begin{array}{c} K=N^{-1}B^{T}L. \end{array}$$

So the state function can be noted as

$$\begin{array}{c} \begin{array}{c} \dot{x}=\left(A-BK\right)x. \end{array} \end{array}$$ (11)

and

$$\begin{array}{c} \begin{array}{c} \dot{\lambda }=-Mx-A^{T}Lx=\dot{L}x+L\dot{x}=\dot{L}x+L\left(A-BN^{-1}B^{T}L\right)x. \end{array} \end{array}$$ (12)

then obtain the following equation by eliminating *x*(*x* ≠ 0)

$$\begin{array}{c} \begin{array}{c} \dot{L}=-LA-A^{T}L+LBN^{-1}B^{T}L-M. \end{array} \end{array}$$ (13)

Because *L*(∞) = *const* or the zero boundary $\dot{L}=0$, the Riccati algebraic equation can be written as

$$\begin{array}{c} \begin{array}{c} -LA-A^{T}L+LBN^{-1}B^{T}L-M=0. \end{array} \end{array}$$ (14)

$$\begin{array}{c} \begin{array}{c} \frac{d}{dt}\left(x^{T}Lx\right)={\dot{x}}^{T}Lx+x^{T}\dot{L}x+x^{T}L\dot{x}. \end{array} \end{array}$$ (15)

The derivation of *x*^*T*^ *Lx* is given by

$$\begin{array}{c} \begin{array}{c} \frac{d}{dt}\left(x^{T}Lx\right)=-x^{T}Mx-u^{T}Nu. \end{array} \end{array}$$ (16)

Upon integrating both sides, we get

$$\begin{array}{c} J=\frac{1}{2}\int_{0}^{\infty }\left(R^{T}MR+u^{T}Nu\right)dt=\frac{1}{2}x{\left(0\right)}^{T}Lx\left(0\right). \end{array}$$

and obtain the minimum functional value, which is

$$\begin{array}{c} J^{*}=\frac{1}{2}x{\left(0\right)}^{T}Lx\left(0\right). \end{array}$$

In addition, we assume that the Lyapunov function is *V*(*x*) = *x*^*T*^ *Px*

$$\begin{array}{c} \dot{V}=-x^{T}Mx-u^{T}Nu<0. \end{array}$$

and it is easy to know it is stable.

Finally, the reaction-diffusion equation with a controller can be written as

$$\begin{array}{c} \frac{\partial }{\partial t}(\begin{array}{c} P\\ Q \end{array})=\left(A-BK\right)(\begin{array}{c} P\\ Q \end{array})+D\nabla^{2}(\begin{array}{c} P\\ Q \end{array}). \end{array}$$ (17)

and

$$\begin{array}{c} \begin{array}{c} \frac{\partial P}{\partial t}=f\left(P,Q\right)+d_{1}\nabla^{2}P+u,\\ \frac{\partial Q}{\partial t}=g\left(P,Q\right)+d_{2}\nabla^{2}Q-u. \end{array} \end{array}$$ (18)

**Fig 2:**
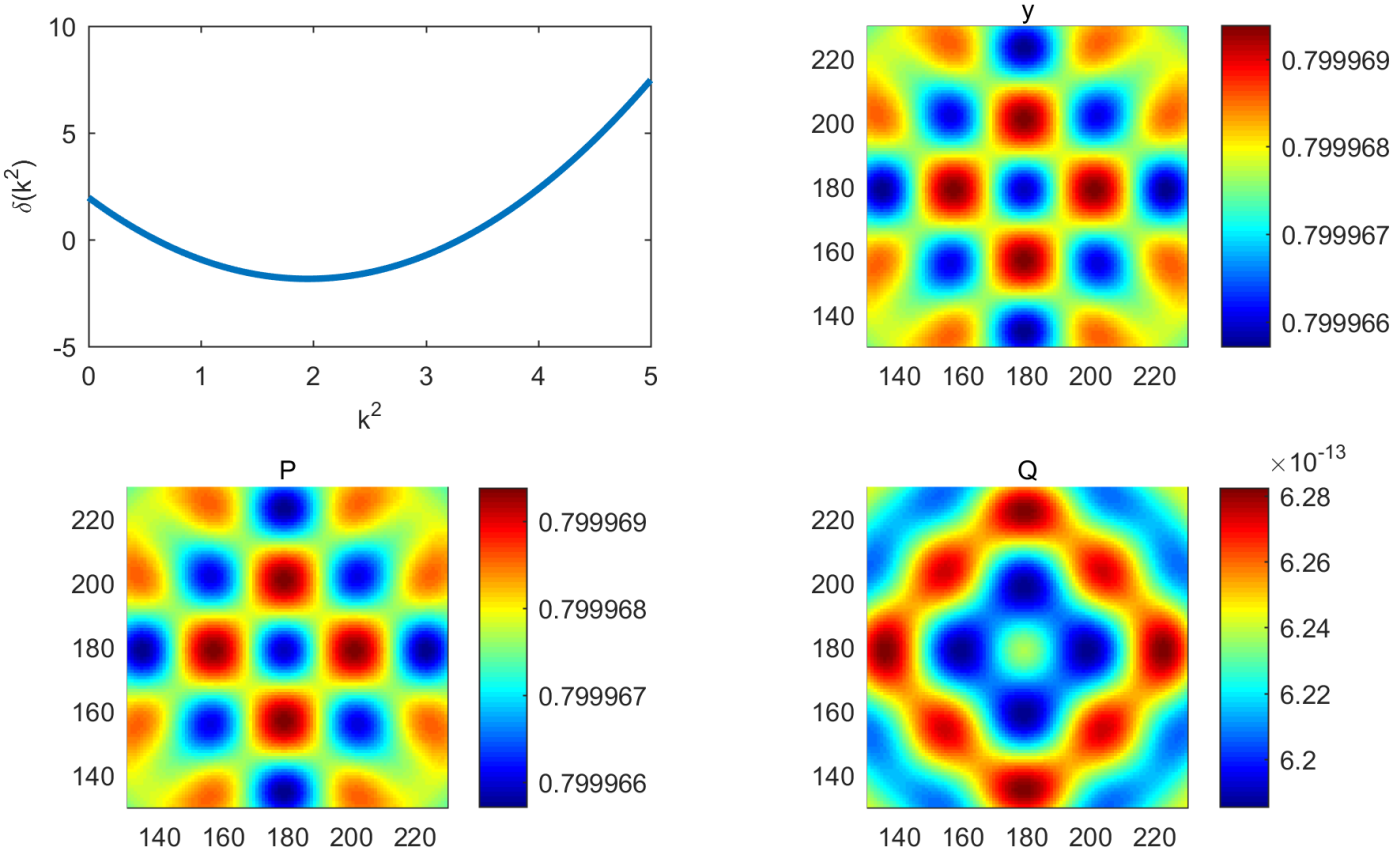
Turing instability without a controller. (a) Dispersion curve indicating the occurrence of Turing instability. (b) Pattern formation of the output (positive) without a controller. (c) Pattern formation of cancer cells without a controller. (d) Pattern formation of immune cells without a controller.

**Fig 3:**
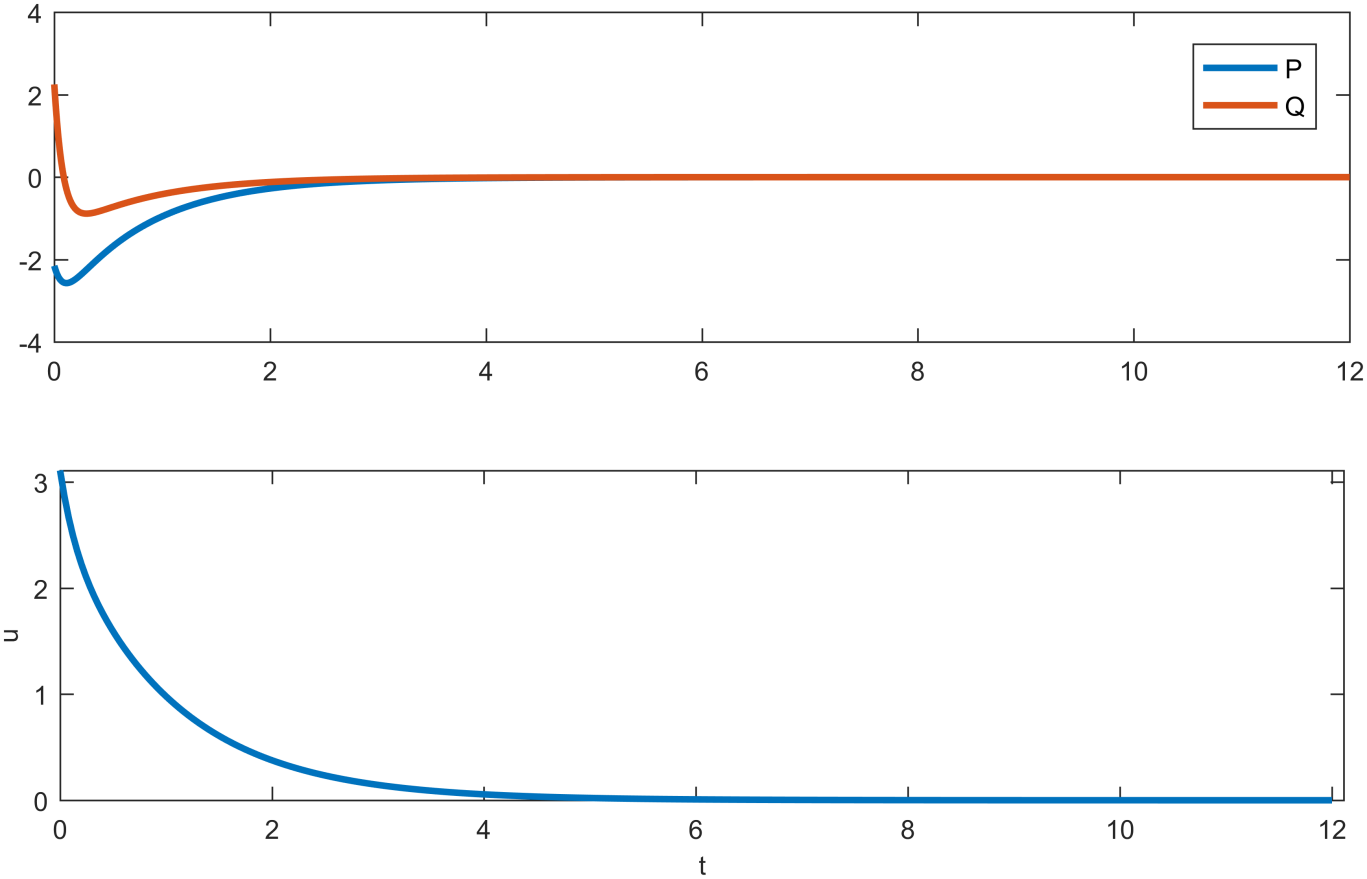
The distribution curves and controller. (a) Cancer cell and immune cell distribution curves for System (5). (b) Curve (controller) showing how much medicine should be given.

**Fig 4:**
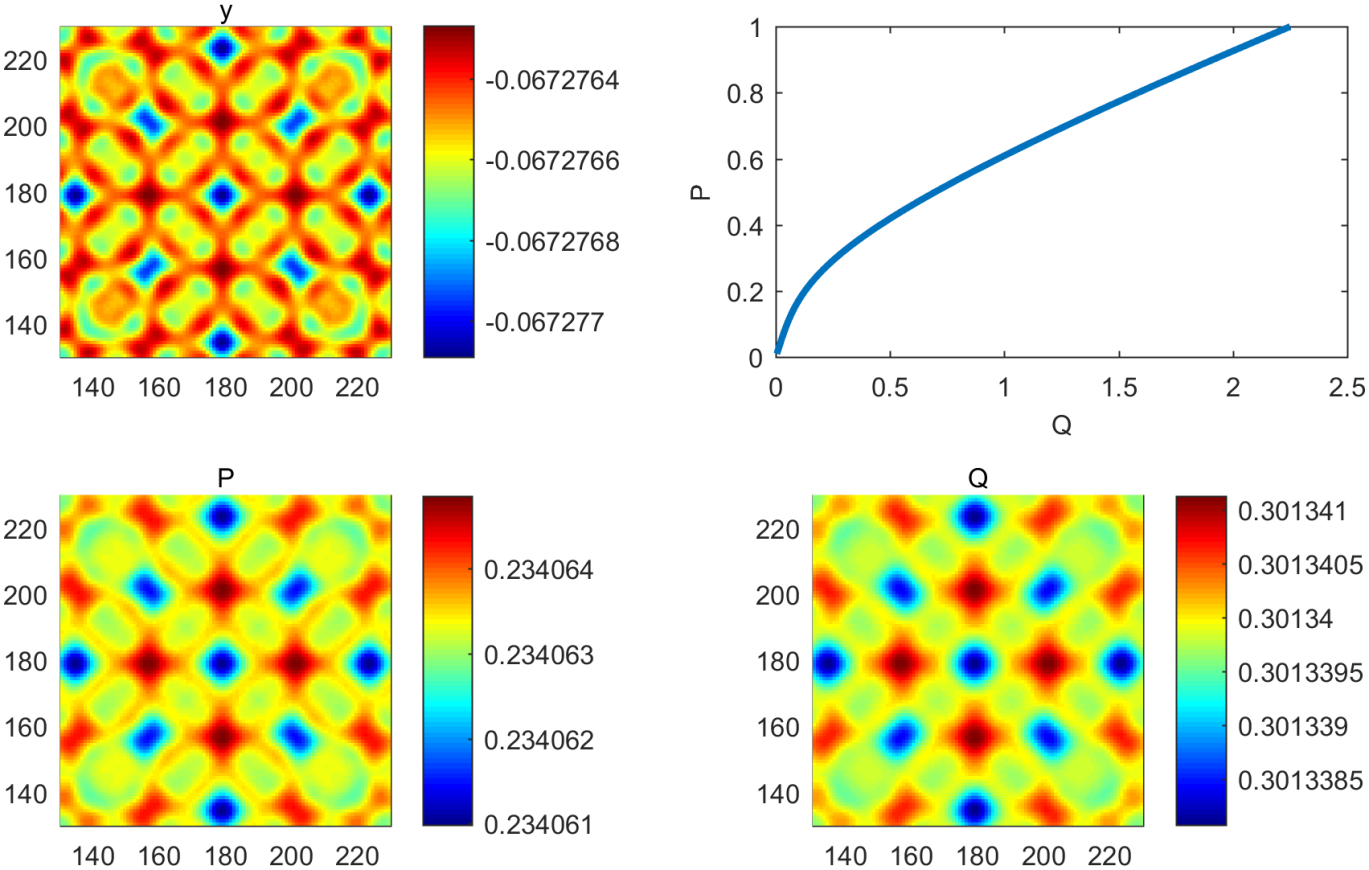
The pattern formation with a controller. (a) Pattern formation of output when the curative medicine is taken. (b) The optimal trajectory. (c) Pattern formation of cancer cells. (d) Pattern formation of immune cells.

**Fig 5:**
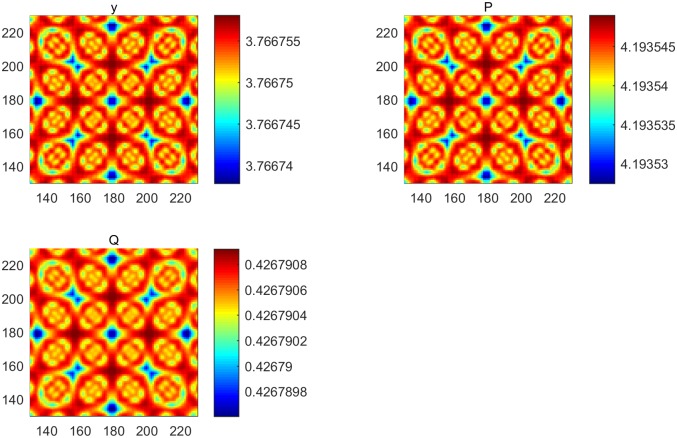
The pattern formation with a controller when *α* = 8. (a) Pattern formation of output. (b) Pattern formation of cancer cells. (c) Pattern formation of immune cells.

**Fig 6:**
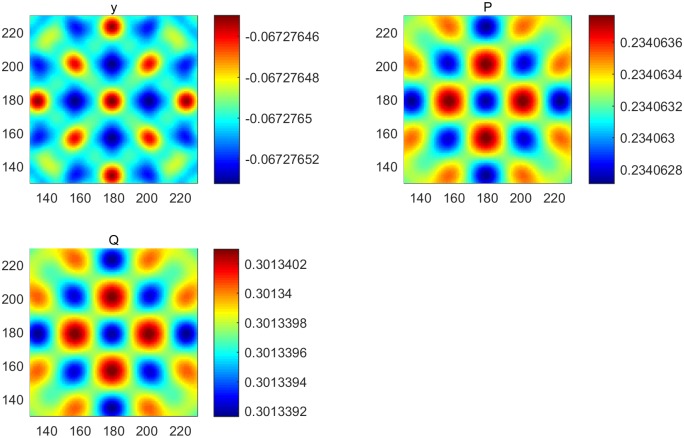
The pattern formation with a controller when *d*_1_ = 2.5. (a) Pattern formation of output. (b) Pattern formation of cancer cells. (c) Pattern formation of immune cells.

The above process is the implementation of control to reaction diffusion that we proposed. We can also validate the result in the following simulation [Figs 2–6] by selecting the appropriate parameter.

## Numerical simulation

In this section, the immune model is simulated numerically by the finite difference method in two spatial dimensions. We set the time step and space step as 0.02 and 1, respectively, and select coefficients of diffusion $d_{1}=\frac{1}{2}$ and *d*_2_ = 2, with each frame being 360 * 360 space units. Assuming that an immune cell can kill a cancer cell and that *y* = *P* − *Q* is an output, we choose parameters *B* = [1 − 1]^*T*^, *C* = [1 − 1] and (*α*, *β*, *γ*, *δ*, *θ*, *η*) = (1.2, 1.5, 2, 2, 1, 2.4). Clearly, the system is the observation. For the first test, we simulate the process from system (4) without a controller. We can thus obtain $k_{c}^{2}=1.95$ and $\delta \left(k_{c}^{2}\right)=-1.8225$, which indicates that the Turing instability and cancer cell cannot be eliminated by an immune cell [Fig 2]. For System (5), we take $k^{2}=k_{c}^{2}$ and obtain *Jmin* = 0.9751 and *K* = [0.9084 − 0.5143]; therefore, *u* = −*Kx* = 0.5143*Q* − 0.9082*P*. The cell density with a controller and the amount of medicine are shown in Fig 3. Pattern formation is also shown in Fig 4, which indicates the effective cure when medicament control is used. In addition, we found that the spot pattern [Fig 5] suggesting a worse outcome when the proliferation of the cancer cells is faster occurs when the diffusion of the cancer cells is faster [Fig 6]. Here, the sign ± is only the representative of density.

## Discussion

Reaction-diffusion system have often been studied [1–20], and more complex patterns involving some biological mechanisms have merged. Understanding how to control these pattern is very important. Therefore, introducing control theory to a reaction-diffusion system is required. Adhesion proteins are known to be essential for the formation of normal epithelial tissues and are tumor suppressors [47]. Cancer cells diffuse easily when the spot pattern is isolated, and normal cells are fixed because the stripe-like pattern links them with each other. In this article, we present systematical analysis of how to control pattern formation under the condition of Turing instability [Fig 2] where the cancer cells are in the ascendancy. Then, we provide a theoretical method to make the immune system effective [Fig 4] using medicine [Fig 3] together with modern control theory, which can be used to cure some diseases. Moreover, by performing a series of numerical simulations, it is found that a system with a regulator has rich spatial dynamics [Figs 2 and 4] and can be treated as the coexistence of cancer cells and immune cells, which can be controlled. However, the pattern formed is the spot pattern[Figs 5 and 6], in which the spot-like terrain is the worst [51]. Although medicine was taken, the condition will worsen when the proliferation and diffusion of cancer cells are faster. Consequently, we provide a way to guide clinical medication for the treatment of some conditions. In addition, this research represents an important advancement in the research of reaction-diffusion equations, especially with regard to the development of nonlinear patterns in complex system. Furthermore, these efforts will improve our understanding of the biological mechanism of pattern formation using control theory in a specific model and will determine the key points to provide possible advice by regulating the main properties of biological systems.
